# Pre-hospital transfusion of plasma in hemorrhaging trauma patients independently improves hemostatic competence and acidosis

**DOI:** 10.1186/s13049-016-0327-z

**Published:** 2016-12-09

**Authors:** Hanne H. Henriksen, Elaheh Rahbar, Lisa A. Baer, John B. Holcomb, Bryan A. Cotton, Jacob Steinmetz, Sisse R. Ostrowski, Jakob Stensballe, Pär I. Johansson, Charles E. Wade

**Affiliations:** 1 Center for Translational Injury Research (CeTIR), Department of Surgery, University of Texas Health Science Center, Houston, TX USA; 2 Section for Transfusion Medicine, Capital Region Blood Bank, Copenhagen University Hospital, Rigshospitalet, Copenhagen, Denmark; 3 Department of Anesthesia, Centre of Head and Orthopedics, Rigshospitalet, Copenhagen University Hospital, Copenhagen, Denmark; 4 Department of Biomedical Engineering, Wake Forest University, Winston Salem, NC 27101 USA

**Keywords:** Pre-hospital, Plasma, RBCs, Trauma, Transfusion, Thrombelastography, Coagulopathy

## Abstract

**Background:**

The early use of blood products has been associated with improved patient outcomes following severe hemorrhage or traumatic injury. We aimed to investigate the influence of pre-hospital blood products (i.e. plasma and/or RBCs) on admission hemostatic properties and patient outcomes. We hypothesized that pre-hospital plasma would improve hemostatic function as evaluated by rapid thrombelastography (rTEG).

**Methods:**

We conducted a prospective observational study recruiting 257 trauma patients admitted to a Level I trauma center having received either blood products pre-hospital or in-hospital within 6 hours of admission. Clinical data on patient demographics, blood biochemistry, injury severity score and mortality were collected. Admission rTEG was conducted to characterize the coagulation profile and hemostatic function.

**Results:**

75 patients received pre-hospital plasma and/or RBCs (PH group; nearly half received both RBCs and plasma) whereas 182 patients only received in-hospital blood products (RBCs, Plasma and Platelets) within 6 hours of admission (IH group). PH patients had lower Glasgow coma scale (GCS) scores, more penetrating injuries, lower systolic blood pressures, lower hemoglobin levels, lower platelet counts and greater acidosis upon ED admission than the IH group (all *p* < 0.05). Despite differences in type of injury and admission vitals indicating that the PH group had more signs of bleeding than the IH group, there were no significant differences in in-hospital mortality (PH 26.7% vs. IH 20.9% p = 0.31). When comparing rTEG variables between PH patients transfused with 0, 1 or 2 units of plasma, more pre-hospital plasma transfusion was tendency towards improved rTEG variables. When adjusting for pre-hospital RBC, pre-hospital plasma was associated with significantly higher rTEG MA (*p* = 0.012) at hospital admission.

**Discussion:**

After adjusting for pre-hospital RBCs, pre-hospital plasma transfusion was independently associated with increased rTEG MA, as well as arrival indices of shock and hemodynamic instability. Besides more severe injury and worse clinical presentation, the group that received pre-hospital transfusion had early and late mortality similar to patients not transfused pre-hospital.

**Conclusions:**

These data suggest that early administration of plasma can provide significant hemostatic and potential survival benefit to severely hemorrhaging trauma patients.

## Background

Death from injury has increased by 20% over the last decade and accounts for more deaths than malaria, tuberculosis and HIV combined [1]. Hemorrhage requiring massive transfusion secondary to trauma remains a major cause of potentially preventable deaths, and development of trauma induced coagulopathy (TIC) further increases the mortality rates of hemorrhaging patients substantially [2]. During the last decade a number of strategies and protocols have been developed and adopted to improve the management and delivery of care to trauma patients with severe hemorrhage. These strategies include: (i) damage control surgery, the use of abbreviated surgery to remedy hemorrhage and contamination with definitive surgery delayed until physiology has been restored [3]; (ii) limited use of crystalloid fluids, which can worsen coagulopathy through dilution of clotting factors and platelets [4]; (iii) hypotensive resuscitation, whereby normalization of blood pressure is delayed until hemorrhage has been surgically controlled [5]; and (iv) balanced blood product use, whereby packed red blood cells (RBCs), plasma and platelets are transfused in ratios of, or near to,1:1:1 mimicking whole blood, which is associated with improved outcomes and may help alleviate TIC [6, 7]. Taken together, these are known as damage control resuscitation (DCR) [8]. As DCR developed, it became clear that early, aggressive resuscitation with blood products could lead to early correction of acidosis, coagulopathy, and hypothermia [9–11].

Rawdan and colleagues reported that moving thawed plasma from the blood bank to the emergency department enabling early balanced blood product transfusions, was associated with a reduction in overall blood product use and a 60% decrease in odds of 30-day mortality [12]. Based on this rapidly evolving experience, the Texas Trauma Institute at Memorial Hermann Hospital (Houston, TX) introduced the availability of two units of O RhD negative RBCs and two units of thawed AB plasma in appropriate temperature controlled containers on four Life Flight (LF) helicopters [13]. Recently, we reported that this novel practice of early hemostatic-resuscitation with blood products was associated with improved hemorrhage-resuscitation and patient outcomes (i.e. improved survival) when compared to trauma patients only receiving crystalloids en route to the hospital [13].

The objective of the present observational study was to investigate the association between pre-hospital administered RBCs and plasma and hemostatic function as evaluated by whole blood thrombelastography (TEG) on arrival at the trauma center as compared to that of patients not receiving pre-hospital blood transfusions. We hypothesized that patients receiving pre-hospital blood products, specifically pre-hospital plasma, would display improved hemostatic function as evaluated by TEG.

## Methods

### Setting and patients

The Texas Trauma Institute at Memorial Hermann Hospital is an American College of Surgeons verified Level I trauma center that is the primary teaching hospital for the University of Texas Health Science Center and operates a hospital-based helicopter program, Life Flight (LF). The helicopters carry two units of type O RBCs and two units of AB plasma for emergent use following established guidelines. This prospective observational cohort study included 257 trauma patients admitted directly from the scene of the injury to the highest level of activation at Memorial Hermann Hospital Texas Medical Center (MHH-TMC) between October 2012 and November 2013. The University of Texas Health Science Center at Houston institutional review board (IRB) approved this study (IRB# HSC-GEN-12-0059).

### Patient selection

All adult trauma patients (≥16 years) who met criteria for full trauma team activation and received blood before arrival at ED (i.e. pre-hospital, PH group) or after hospital admittance within 6 h of ED arrival (i.e. in-hospital, IH group) were included in this study.

The criteria for exclusion were: pregnancy, prisoner, had a burned body surface area >20% or enrolled in another study. Patients from whom we could not obtain a blood draw were excluded from analysis. Informed consent was obtained from the patient or a legally authorized representative within 72 h of admission. If the patients were discharged or died within 24 h, a waiver of consent was granted. In cases which consent could not be obtained, the patient was excluded from the study, and their blood samples were destroyed. Patient information on baseline demographics, clinical, physiologic, and laboratory tests were extracted from patient records and the institutional trauma registry.

### Sample collection

Blood samples were collected immediately upon hospital admission on all adult trauma patients (≥16 years) who met criteria for full trauma team activation i.e., PH patients had samples drawn after PH blood transfusion whereas IH patients had samples drawn before any blood transfusion. In all cases, blood was drawn into citrated tubes for conducing the rapid-TEG assay.

### Rapid Thrombelastography (rTEG)

All rTEG specimens were run on a thrombelastograph 5000 (Haemoscope Corporation, Niles, IL). Admission rTEGs were performed using the accepted citrate reversal method. Specimens were collected in a small (3 mL) citrated tube, transported to the Emergency Department Stat Laboratory. The citrate was immediately reversed by adding calcium chloride according to the recommendations of the manufacturer within the rTEG package insert. After this, TEG was performed within 5–10 min of blood sampling, using tissue factor and kaolin as activators. Staff laboratory technicians in the Memorial Hermann Hospital Emergency Department performed all the rTEG assays during the defined study period. These same technicians performed all the quality controls on the TEG analyzers, doing so every 8 h. Quality control was performed per the package insert from the manufacturer.

The rTEG generates several values that describe the hemostatic process: The first value generated is the activated clotting time (ACT), which is the time in seconds between initiation of the test and the initial fibrin formation (normal range, 86–118 s). Similar to the ACT, the r-value (also known as the reaction time (R-time), 0–1 min) expresses the time between the start of the assay and beginning of clot formation. The K-time (normal range, 1–2 min) is the time needed to reach 20-mm clot strength and the alpha (α) angle (normal range, 66–82°) determines the slope of the tracing that represents the rate of clot formation. The maximal amplitude (MA; normal range, 54–72 mm) is the greatest amplitude of the tracing and the *G*-value (normal range, 5,300–12,000 dynes/cm^2^) is a global measure of absolute clot strength (both enzymatic and platelet contributions) and is decreased in hypocoagulable states. LY30 (normal range, 0.0–7.5%) is the percent amplitude reduction at 30 min after MA, illustrating the amount of fibrinolysis.

### Statistics

Statistical analysis was performed using STATA (IC version 12.1, College Station, TX) and SPSS 22 (IBM Corporation, New York, US). Descriptive data are presented as medians with inter quartile ranges (IQR) or as % (proportions).

Non-parametric statistical tests (Mann-Whitney *U* test and Kruskal-Wallis) and Chi-square tests were used to evaluate group differences (PH vs. IH groups), as appropriate. *P*-values <0.05 were considered significant.

To investigate the association of pre-hospital RBCs and plasma on admission for rTEG MA, a parameter with significant predictive value for transfusion requirements and mortality [14–16], we conducted a linear regression analysis. Univariate and multivariate linear regression models were constructed for each group (PH, IH) and combined including both groups. Multivariate linear regression models were adjusted for admission vital differences between the PH and IH groups (systolic blood pressure (SBP), hemoglobin, platelet count and pH) as well as adjusting for mode of transport (i.e. LF or ambulance) and amount of PH-RBC and PH-Plasma in the model including both groups (PH and IH). Furthermore we compared rTEG parameters in PH patients receiving plasma based on the amount administered (0, 1 and 2 units plasma) by ANOVA and Tukey post-hoc test.

## Results

### Patient characteristics

A total of 257 patients were included in the study, 75 (29%) patients received pre-hospital (PH) blood, whereas 182 (71%) patients only received blood in-hospital (IH) within 6 h of hospital admission. Eighty-six patients were transported by ground ambulance and 171 patients by LF, of which 44% received a PH blood transfusion (Table 1). The median age of the study cohort was 37 years, 81% were male, with a median injury severity score (ISS) of 26 (Table 1). Patients in the PH group demonstrated a higher incidence of penetrating injuries (45 vs. 25%, *p* = 0.002), field intubations (39 vs. 21%, *p* = 0.005), positive FAST exams and presented with lower GCS scores compared to patients in the IH group (Table 1). Additionally, patients receiving PH blood displayed significantly lower systolic blood pressure (SBP), hemoglobin, platelet count and more acidosis than IH, suggesting a more pronounced degree of hemorrhage and/or shock in the PH group. Lastly, patients receiving PH blood received more units of blood products within 6 and 24 h after admission (Table 1). Despite differences in type of injury and admission vitals indicating more severe injury and bleeding in the PH group, there were no significant differences in mortality (Table 1).

**Table 1:** Patient demographics, injury severity, admission vitals, transfusion and outcome in 257 adult trauma patients

	Units	Overall (*n* = 257)	PH (*n* = 75)	IH (*n* = 182)	*P*-value
Age	Years	37 (25, 53)	34 (23, 53)	39 (26, 53)	0.259
Male	n (%)	208 (80.9%)	62 (82.7%)	146 (80.2%)	0.650
Injury Scores and severity					
ISS	Score	26 (17, 34)	29 (17, 41)	26 (17, 34)	0.106
GCS	Score	10 (3, 15)	3 (3, 15)	12 (3, 15)	**0.022**
Positive FAST	%	54 (31.4%)	26 (48.1%)	28 (23.7%)	**0.001**
Penetrating Injury	%	80 (31.1%)	34 (45.3%)	46 (25.3%)	**0.002**
Intubated in the field	%	63 (27.2%)	29 (39.2%)	34 (21.5%)	**0.005**
Admission Vitals					
SBP	mmHg	98 (80, 123)	90 (77, 113)	100 (80, 125)	**0.044**
DBP	mmHg	60 (48, 74)	59 (50, 69)	60 (48, 76)	0.299
Heart rate	Bpm	108 (88, 130)	111 (90, 133)	108 (85, 130)	0.425
Hemoglobin	g/dL	12.9 (11.3, 14.2)	12.4 (11.3, 13.4)	13.1 (11.35, 14.3)	**0.011**
Platelet count	x10^3^/μL	215 (163, 250)	193 (152, 223)	225 (174, 257)	**0.001**
pH		7.26 (7.15, 7.32)	7.21 (7.06, 7.32)	7.27 (7.18, 7.33)	**0.002**
Base Excess	mEq/L	−5 (−10, −2)	−6 (−10, −3)	−4 (−10, −1)	0.073
Mode of Transport					
LF	%	171 (66.8%)	75 (100%)	96 (52.7%)	**<0.001**
Transfusion: Prehospital					
RBC	Units	0 (0, 0)	1 (1, 2)	0 (0, 0)	**NA**
Plasma	Units	0 (0, 0)	1 (1, 2)	0 (0, 0)	**NA**
Transfusion: Admission - 6 h total transfusions					
RBC	Units	4 (2, 10)	8 (4, 14)	3 (1, 8)	**<0.001**
Plasma	Units	4 (2, 8)	6 (3, 12)	3 (2, 7)	**<0.001**
Platelets	Packs	0 (0, 6)	6 (0, 12)	0 (0, 6)	**<0.001**
Transfusion: Admission - 24 h total transfusions					
RBC	Units	5 (2, 12)	10 (4, 15)	4 (2, 9)	**<0.001**
Plasma	Units	4 (2, 9)	7 (3, 14)	4 (2, 8)	**<0.001**
Platelets	Packs	6 (0, 12)	6 (0, 18)	0 (0, 6)	**<0.001**
Outcomes					
6-h mortality	%	25 (9.7%)	10 (13.3%)	15 (8.2%)	0.210
24-h mortality	%	31 (12%)	12 (16%)	19 (10.4%)	0.213
In-hospital mortality	%	58 (22.6%)	20 (26.7%)	38 (20.9%)	0.313

Patients met criteria for full trauma team activation and either received blood before arrival at ED (PH) or in the first 6 h after hospital admission (IH). Medians (IQR) or *n* (%) are reported

All the bold numbers reflect data that is significant

### Pre-hospital Plasma is associated with improved hemostasis

Despite the variations between the two groups, no significant differences were found in the median rTEG values, respectively for the PH vs. IH, for ACT (121 vs. 121), R-time (0.8 vs. 0.8), K-time (1.65 vs. 1.45), Angle (70 vs. 73) and Ly30 (1 vs. 1.4), upon arrival at the hospital. However, at hospital admission PH displayed significant lower median rTEG MA (62 vs. 64, *P* = 0.020) and *G*-value (8.1 vs. 8.69, *P* = 0.009) compared to IH patients. Table 2 shows the distribution of pre-hospital blood products transfused. Nearly half of the PH group received 1 unit of RBCs and plasma each (Table 2). When comparing rTEG values ACT, R-time, K-time, α-Angle, MA, G and LY30 in PH patients receiving 0, 1 and 2 units of plasma pre-hospital unadjusted for PH RBC, patients receiving 2 units of plasma pre-hospital had the highest mean values of rTEG α-Angle (70.57 vs. 67.51 vs. 72.00° for plasma = 0, 1 and 2 units, respectively), rTEG MA (58.73 vs. 57.30 vs. 62.55 mm for plasma = 0, 1 and 2 units, respectively) and rTEG G (5.93 vs. 7.36 vs. 8.41 dyn/cm^2^ for plasma = 0, 1 and 2 units, respectively). Furthermore, patients receiving 2 units of plasma pre-hospital had lower average values of K-time (1.63 vs. 2.07 vs. 1.59 min for plasma = 0, 1 and 2 units, respectively) and R-time (2.07 vs. 1.07 vs. 0.72 min for plasma = 0, 1 and 2 respectively). There was a significant difference in R-time between patients receiving no pre-hospital plasma compared to patients receiving 2 units of plasma (*P* = 0.023).

**Table 2:** Distribution of patients receiving pre-hospital blood products

		Plasma			
		0 U	1 U	2 U	Total RBC
	0 U	0 (0%)	10 (13%)	3 (4%)	13 (17%)
RBC	1 U	2 (3%)	30 (40%)	7 (9%)	39 (52%)
	2 U	1 (1%)	2 (3%)	20 (27%)	23 (31%)
	Total Plasma	3 (4%)	42 (56%)	30 (40%)	75 (100%)

### Predictors of hemostatic competence

In the PH group, the unadjusted univariate linear regression demonstrated that pre-hospital plasma was associated with significantly higher rTEG MA values (one unit increase in PH plasma was associated with (β coefficient) 13.95 mm, 95%CI [3.13, 24.77], *p* = 0.012) higher MA at hospital admission in a model only adjusting for PH RBC. The interaction between PH RBC and plasma did not appear to have a significant effect on admission rTEG MA values (data not shown). When adjusting for pH, hemoglobin, platelet count, SBP, and in PH patients PH-RBC and PH-Plasma, higher pH and higher platelet count were independent predictors of rTEG MA in IH patients and higher pH, higher platelet count and PH-Plasma were associated with higher rTEG MA in PH patients (all *p* < 0.05, data not shown).

When conducting the analysis non-stratified (including both PH and IH groups in the same analysis), and instead adjusting for transportation (by LF or ambulance) and admission vital differences, and PH transfusion with Plasma and RBC, PH plasma transfusions were associated independently with higher rTEG MA values together with higher pH, higher SBP and higher platelet count (Table 3).

**Table 3:** Admission rapid thrombelastography (rTEG) MA-value evaluated by linear regression in 257 adult trauma patients

Variable	Units	Univariate		Adjusted	
		β (95% CI)	*P* value	β (95% CI)	*P* value
LF	Yes	−0.52 (−3.25, 2.21)	0.709	1.02 (−1.41, 3.46)	0.408
pH	mEq/l	30.00 (21.53, 38.47)	**<0.001**	23.55 (15.26, 31.8)	**<0.001**
Hemoglobin	g/dl	0.80 (0.19, 1.42)	0.011	−0.12 (−0.68, 0.43)	0.666
Platelet counts	10^3^/μL	0.07 (0.05, 0.08)	**<0.001**	0.06 (0.04, 0.08)	**<0.001**
SBP	mmHg	0.07 (0.04, 0.11)	**<0.001**	0.04 (0.00, 0.07)	**0.047**
PH-RBC	units	−1.89 (−3.86, 0.09)	0.061	−1.82 (−4.82, 1.18)	0.232
PH-Plasma	units	−0.85 (−2.68, 0.99)	0.364	2.88 (0.10, 5.66)	**0.042**

Regression coefficients (β) with 95% confidence intervals (95% CI) and *p*-values are displayed. *P*-values <0.05 are shown in bold. Predicted change in rTEG MA (mm) associated with: LF (yes) and one unit higher pH, Hemoglobin, Platelet count, SBH, PH-RBC and PH-Plasma

## Discussion

This study found that after adjusting for pre-hospital RBCs, pre-hospital plasma transfusion was independently associated with increased rTEG MA, as well as arrival indices of shock and hemodynamic instability. Acidosis and low platelet count were found to impede hemostatic potential evaluated by rTEG. Besides more severe injury and worse clinical presentation, the group that received pre-hospital transfusion had early and late mortality similar to patients not transfused pre-hospital.

Reduced clot strength, as evaluated by TEG, has repeatedly been associated with increased bleeding, transfusion requirements and mortality in severely injured trauma patients [16, 17]. In the present study, plasma transfusion PH was independently associated with higher rTEG MA values, also after adjusting for pre-hospital RBCs. Furthermore, patients in the PH group receiving the highest amount of pre-hospital plasma transfusions differed in their hemostatic competence as evaluated by rTEG when compared to the patients in the PH group only receiving one or zero pre-hospital plasma. Given that the most severely injured and bleeding patients are expected to receive the highest amount of plasma pre-hospital, this suggests that pre-hospital plasma is more beneficial than pre-hospital RBCs for improving hemostasis in the acute/pre-hospital setting, in accordance with the coagulation factor and fibrinogen content in plasma compared to RBC.

Furthermore, PH patients were significantly different from IH patients. PH patients presented with more penetrating injuries, more positive FAST exams, lower systolic blood pressures, lower hemoglobin, lower platelet count, lower pH upon arrival to the trauma bay and lower rTEG MA and G, despite receiving pre-hospital RBC and/or plasma transfusions during transport. However, 52.7% of the IH patients were transported by LF where blood products are available; indicating that the prehospital blood transfusion by LF was not deemed necessary for these patients as they were not transfused. Furthermore, IH patients only received three RBCs and three plasma units within six hours of hospital admission, which suggests only moderate hemorrhage compared to eight RBCs and six plasma units in the PH group. Due to these differences, we stratified by group as well as conducting adjusted regression analyses. Regardless of group, however, acidosis and low platelet count were the strongest predictors of reduced hemostatic competence evaluated by rTEG MA values. This indicates that future analyses of rTEG should consider adjusting for pH and platelet count to make appropriate comparisons.

In addition, the acid buffer capacity of plasma is known to be 25–50 times better than crystalloids solutions [18], and despite the divergence between the two groups, the data shows no significant difference in the 6-h, 24-h, and the overall mortality. This indicates that pre-hospital plasma transfusion may lead to early correction of acidosis thereby mitigating coagulopathy, which potentially can be translated into improved survival though this has to be investigated in future studies designed specifically to answer this question.

The study has several limitations. First, it should be noted that we could not obtain blood samples from “in-the-field”, prior to when the patient received pre-hospital transfusions. As such, we cannot definitively assess the effect of each transfusion and potential changes in the TEG values from “in-the-field” vs. hospital admission. Future studies are needed to evaluate these prehospital and pre-transfusion acute changes in clotting properties. Furthermore, another limitation is the relatively small sample size of 75 patients within the PH group and nearly half of these patients received one unit of RBCs and plasma pre-hospital. This emphasize the need for future studies investigating the relative benefits of adding RBCs to plasma in a larger number of patients, preferably sampling blood for pre-hospital analysis.

## Conclusion

In conclusion, after adjusting for pre-hospital RBCs, pre-hospital plasma transfusion was independently associated with higher rTEG MA. Acidosis (low pH levels) and low platelet counts were the strongest predictors of poor hemostasis evaluated by rTEG. Besides, more severe injury and worse clinical presentation, the PH group had early and late mortality similar to the IH group. Overall, these data imply that early administration of plasma may have beneficial effects on improving hemostasis, which may potentially translate into improved patient survival in future studies with larger sample sizes.

## References

[1] Norton R, Kobusingye O (2013). Injuries. N Engl J Med.

[2] Johansson PI, Ostrowski SR, Secher NH (2010). Management of major blood loss: an update. Acta Anaesthesiol Scand.

[3] Rotondo MF, Schwab CW, McGonigal MD, Phillips GR, Fruchterman TM, Kauder DR (1993). Damage control: an approach for improved survival in exsanguinating penetrating abdominal injury. J Trauma.

[4] Cotton BA, Guy JS, Morris JA, Abumrad NN (2006). The cellular, metabolic, and systemic consequences of aggressive fluid resuscitation strategies. Shock.

[5] Bickell WH, Wall MJ, Pepe PE, Martin RR, Ginger VF, Allen MK (1994). Immediate versus delayed fluid resuscitation for hypotensive patients with penetrating torso injuries. N Engl J Med.

[6] Duchesne JC, Hunt JP, Wahl G, Marr AB, Wang YZ, Weintraub SE, et al. Review of current blood transfusions strategies in a mature level I trauma center: were we wrong for the last 60 years? J Trauma. 2008;65(2):272–6. discussion 6–8. 10.1097/TA.0b013e31817e5166 18695461

[7] Borgman MA, Spinella PC, Perkins JG, Grathwohl KW, Repine T, Beekley AC (2007). The ratio of blood products transfused affects mortality in patients receiving massive transfusions at a combat support hospital. J Trauma.

[8] Hodgetts TJ, Mahoney PF, Kirkman E (2007). Damage control resuscitation. J R Army Med Corps.

[9] Holcomb JB, Jenkins D, Rhee P, Johannigman J, Mahoney P, Mehta S (2007). Damage control resuscitation: directly addressing the early coagulopathy of trauma. J Trauma.

[10] Morrison JJ, Ross JD, Poon H, Midwinter MJ, Jansen JO (2013). Intra-operative correction of acidosis, coagulopathy and hypothermia in combat casualties with severe haemorrhagic shock. Anaesthesia.

[11] Duchesne JC, Islam TM, Stuke L, Timmer JR, Barbeau JM, Marr AB (2009). Hemostatic resuscitation during surgery improves survival in patients with traumatic-induced coagulopathy. J Trauma.

[12] Radwan ZA, Bai Y, Matijevic N, del Junco DJ, McCarthy JJ, Wade CE (2013). An emergency department thawed plasma protocol for severely injured patients. JAMA Surg.

[13] Holcomb JB, Donathan DP, Cotton BA, Del Junco DJ, Brown G, Wenckstern TV (2015). Prehospital Transfusion of Plasma and Red Blood Cells in Trauma Patients. Prehosp Emerg Care.

[14] Holcomb JB, Minei KM, Scerbo ML, Radwan ZA, Wade CE, Kozar RA (2012). Admission rapid thrombelastography can replace conventional coagulation tests in the emergency department: experience with 1974 consecutive trauma patients. Ann Surg.

[15] Johansson PI, Sorensen AM, Larsen CF, Windelov NA, Stensballe J, Perner A (2013). Low hemorrhage-related mortality in trauma patients in a Level I trauma center employing transfusion packages and early thromboelastography-directed hemostatic resuscitation with plasma and platelets. Transfusion.

[16] Nystrup KB, Windelov NA, Thomsen AB, Johansson PI (2011). Reduced clot strength upon admission, evaluated by thrombelastography (TEG), in trauma patients is independently associated with increased 30-day mortality. Scand J Trauma Resusc Emerg Med.

[17] Plotkin AJ, Wade CE, Jenkins DH, Smith KA, Noe JC, Park MS (2008). A reduction in clot formation rate and strength assessed by thrombelastography is indicative of transfusion requirements in patients with penetrating injuries. J Trauma.

[18] Traverso LW, Medina F, Bolin RB (1985). The buffering capacity of crystalloid and colloid resuscitation solutions. Resuscitation.

